# A New All-Season Passive Sampling System for Monitoring H_2_S in Air

**DOI:** 10.1100/tsw.2002.87

**Published:** 2002-01-18

**Authors:** Hongmao Tang, James Sandeluk, Linda Lin, J. WIlliam Lown

**Affiliations:** Centre for Passive Sampling Technology, Maxxam Analytics, Inc., 9331-48 Street, Edmonton, AB, Canada T6B 2R4,; Department of Chemistry, University of Alberta, Edmonton, AB, Canada T6G 2G2,

**Keywords:** passive sampler, diffusion, air pollution, hydrogen sulfide

## Abstract

A new Maxxam All-Season Passive Sampling system for monitoring H2S in air has been developed. This passive sampling system employs the same approaches as the Maxxam All-Season Passive Sampling Systems for monitoring SO_2_, NO_2_, and O_3_ reported previously. This system has been extensively tested in the lab (temperature from -20 to 20°C, relative humidity from 30 to 84%, and wind speed from 0.5 to 150 cm/s) and validated in field studies. Comparing measurements obtained with the use of the new passive sampling system with equivalent measurement with the use of an active filter pack H2S sampler yielded an accuracy of greater than 85%. The new H_2_S passive sampling system can be used to measure ambient H_2_S concentrations ranging from 0.02 to 7 ppb based on a 1-month exposure period. There is no significant interference found from other sulfur compounds in air. This system has been used in many air monitoring projects.

### INTRODUCTION

Hydrogen sulfide is a colorless gas with a characteristic rotten egg odor. It is a toxic and malodorous air pollutant. It has adverse effects on lead-based paints and is corrosive to certain metals. Concerns are frequently voiced by both scientists and the public about health problems that may be caused by exposure to low levels of $H_2S$ in air. $H_2S$ is produced both naturally and through industrial processes, such as from the burning of fuels, sulfur recovery processes, oil refining, etc. Regulations require the monitoring of $H_2S$. Over the past few decades, many sampling methods for monitoring $H_2S$ in ambient air have been developed and subsequently improved[1,2,3,4,5,6,7]. These methods can generally be classified as either active or passive.

Active methods are generally accurate since air is directly pumped through collection devices to sampler air pollutants. But electrical power is required to operate an active sampler. The operation of an active real-time sampler, such as an $H_2S$ analyzer, is more complicated. Electricity is not the only requirement; road, shelter, an air conditioner, and so on are also needed.

A passive (or diffusive) sampler is a device which is capable of taking samples of gas or vapor pollutants from the atmosphere at a rate controlled by a physical process such as diffusion through a static air layer or permeation through a membrane. However, a passive sampler does not require the active movement of the air through the sampler. Passive samplers are generally simple in construction and do not require electricity. Therefore, passive samplers are more cost effective and convenient to use than active samplers.

The major difference between active and passive samplers is the fact that the active sampler has a known sampling rate, which is the pump's flow rate. The passive sampler's sampling rate depends on many factors, such as temperature, relative humidity (RH), wind direction, wind speed (WSP), sampler design, collection media, etc. If a passive sampler is to be used in all climatic conditions, there must be a reasonable way to calculate the sampling rate. It would be highly unreasonable to expect that a passive sampler's sampling rate would not vary from −30 to +30$^{o}$C and from 90 to 15% RH. Therefore, the key factor for using passive samplers is how to determine their sampling rates.

An $H_2S$ passive sampler, which has been in common use for many years, utilizes a paper impregnated with zinc acetate[1]. The detection limit of $H_2S$ by using this method is quite high (2 mg/l in the test solution), and interference from $SO_2$ in the atmosphere is inevitable. Therefore the accuracy for this method is questionable. Like other inadequate passive methods, the zinc acetate paper is directly exposed to the atmosphere without any protection such as a diffusion barrier. The diffusion barrier serves to eliminate the effects of particulate contamination and also makes it possible to account for the variables that affect the sampling rate of the device. Several other groups have studied different types of $H_2S$ passive samplers[3,4,5,6,7]. The major problem with these reports is still related to a given fixed sampling rate, which does not reflect the interference from changes in meteorological data.

With the support of Alberta Environmental Protection (AEP), the Alberta Research Council (ARC), the Clean Air Strategic Alliance of Alberta (CASA), and the National Research Council of Canada (NRC), the Centre for Passive Sampling Technology of Maxxam Analytics, Inc. (CPSTM) has successfully developed an All-Season Passive Air Sampling System (PASS) for sampling $SO_2$, $NO_2$, and $O_3$ in the atmosphere[8,9,10]. A description of the special features of the PASS follows.

- A new passive sampler body and a rain shelter were designed that allow the passive sampler to be installed face downwards outdoors. This design helps the PASS avoid dust problems and facilitates air movement parallel to the diffusion barrier surface (Fig. 1).
- An equation that accounts for variations in temperature, RH, and WSP is used to calculate sampling rates.
- The PASS is very sensitive. It can be used to collect sub ppb levels of $SO_2$, $NO_2$, and $O_3$ in air.
- The collection time is very flexible. The PASS can be exposed from 4 h up to 6 months, depending on the monitored pollutant and its average concentration in air.
- By attaching a clip to the sampler body, the PASS can be used as a personal monitor.

The downward-facing installation of the passive samplers can prevent the precipitation of suspended particles in the atmosphere on the surface of the diffusion barriers. This prevents the air pollutants from being absorbed by the particles and avoids the pore-size change of the diffusion barrier, which will eventually change the sampling rate. The downward-facing installation also helps to obtain air movement across the diffusion barrier surface of a passive sampler.

The PASS for $SO_2$, $NO_2$, and $O_3$ has been independently validated by ARC in 1998 (supported by NRC) (see Fig. 2) and AEP from 1998 to 1999. Very encouraging results have been obtained[11,12].

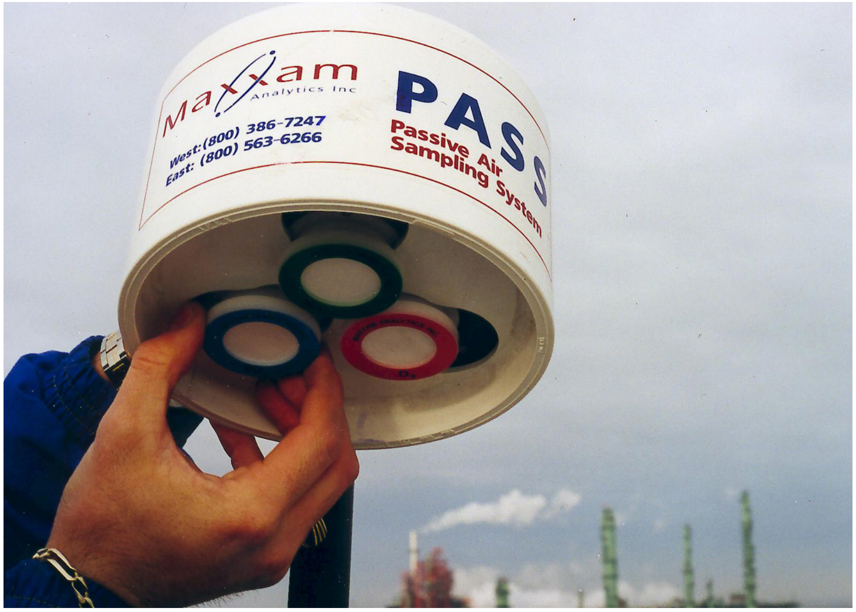

**FIGURE 1.** Maxxam All-Season Passive Air Sampling System.

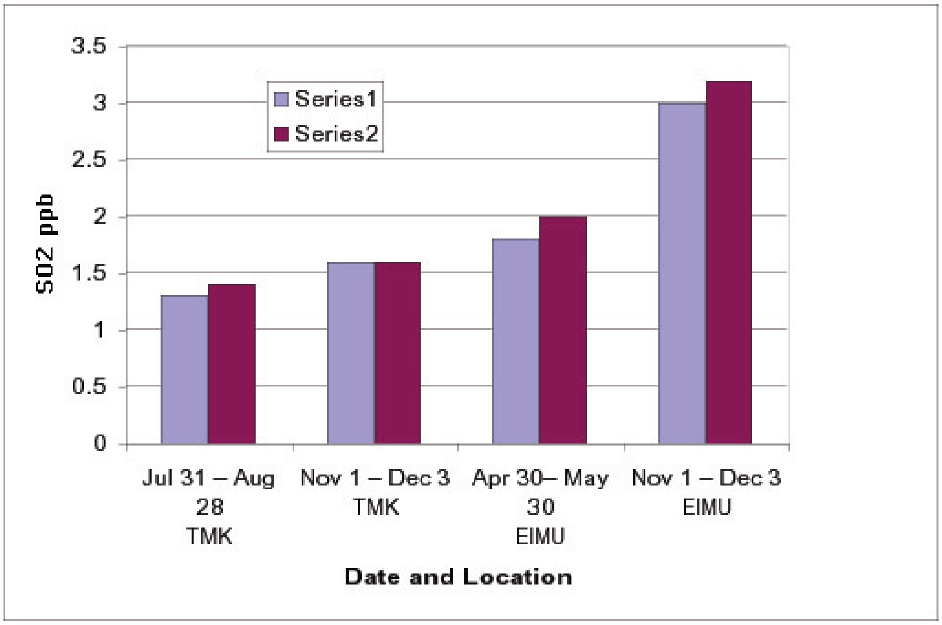

**FIGURE 2.** Field validation of Maxxam All-Season $SO_2$ Passive Sampling System by ARC. EIMU and TMK are locations in Alberta, Canada.

In this paper, a new Maxxam All-Season Passive Sampling System for $H_2S$ (PASS-HS) is reported. The PASS-HS employed the same approaches as the PASS for $SO_2$, etc.[8,9,10]. Laboratory and field studies have proved that the PASS-HS is a reliable device for monitoring $H_2S$ in air. The PASS-HS was first studied in a chamber at different climates. An equation associated with temperature, RH, and WSP was derived from chamber studies.

The PASS-HS has been validated for several months in many Canadian locations under different climatic conditions. The equation from laboratory studies was used to calculate sampling rates in each location. The passive sampler results were compared with a colocated active filter pack sampler. Based on 1-month exposure, the PASS-HS can be used to measure ambient $H_2S$ concentrations ranging from 0.02 to 7 ppb. The accuracy is 85% higher than the active filter pack sampler.

### EXPERIMENTAL

#### Maxxam All-Season $H_2S$ Passive Sampling System (PASS-HS)

The PASS-HS includes four parts: the passive sampler, the diffusion barrier, the collection filter, and the rain shelter. An equation associated with meteorological variables is used to calculate sampling rates. The equation is derived from the design of the passive sampler, the rain shelter, the diffusion barrier, and the collection filter.

##### *PASS-HS Passive Sampler*

A picture of the PASS-HS passive sampler is shown in Fig. 3. The $H_2S$ passive sampler body has the same dimensions as the PASS $SO_2$ reported in Tang et al.[8]. It contains a diffusion barrier, a collection filter which is coated with silver nitrate–based compounds, and an air gap between the diffusion barrier and the collection medium as a diffusion zone. There is an edge at the bottom of the passive sampler, which is suitable for downward-facing installation of the passive sampler in the rain shelter. The diffusion barriers and collection filters are commercially available at Maxxam Analytics, Inc.

##### *PASS-HS Rain Shelter*

A picture of the PASS-HS rain shelter is shown in Fig. 1. Triplicate passive samplers could be installed in the plate facing downwards. An outside bracket was used to fasten the shelter to fittings.

#### $H_2S$ Active Filter-Pack Sampler

An active filter-pack $H_2S$ sampler (AFHS) was employed in this study[13]. The same collection filters used in the PASS-HS are installed in the AFHS for collecting $H_2S$ in air. Three filters were installed in the AFHS in order to prevent any breakthrough of $H_2S$. A personal pump (Model 224-PCXR3, SKC, USA) was used to withdraw air through the AFHS.

#### Laboratory Study

##### *Test Procedure*

The PASS-HS was first tested at our laboratory, using a laboratory system that has been reported[8,9,10] to measure sampling rates at different temperatures, humidities, and wind speeds. The major components of the system were as follows:

1. A permeation oven to generate known $H_2S$ concentrations (Model 8861, Bendix Corporation, Lewisburg, WV).
2. A humidity control system to maintain the specified RH. This device was made from a 4-l Erlenmeyer flask.

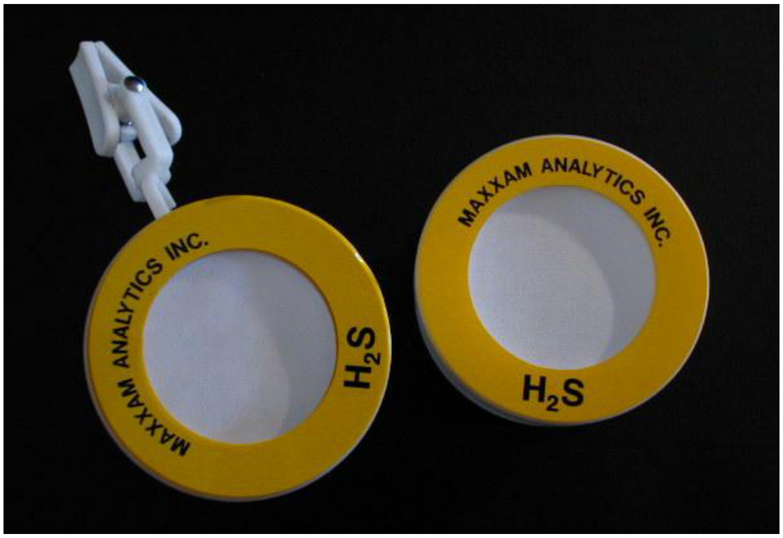

**FIGURE 3.** Maxxam $H_2S$ Passive Sampler. The left sampler with a clamp can be used for personal exposure.

3. An $H_2S$ analyzer to continuously monitor $H_2S$ concentrations during the course of the experiment (TECO Model 45C, Thermo Environmental Instruments Inc., Franklin, MA).
4. Temperature (Fluke Model 80TK and 80T-150U, John Fluke Mfg. Co., In., Everett, WA) and humidity (Vaisala Probe CS500, Vaisala Inc., Woburn, MA) probes to continuously monitor those variables.
5. An exposure chamber in which the passive samplers could be mounted.

The $H_2S$ passive samplers were exposed in the chamber at different conditions. The $H_2S$ concentrations ranged from 6 to 50 ppb, the temperatures from –20 to 20°C, the RH from 30 to 84%, and WSP from 0.5 to 150 cm/s. Triplicate passive samplers and duplicated blanks were studied. The exposure period was about 24 h. The $H_2S$ will be collected as $Ag_2S$ in the collection media.

After exposure, the media in the exposed and blank passive samplers were extracted with an extraction solution, 0.1 mole sodium hydroxide and sodium cyanide (Fisher Scientific, Nepean, CA, purified grade). Quantification is achieved by the selective quenching effects of sulfide ($S^{-2}$) on the fluorescence of fluorescein mercury acetate (FMA) measured by a filter fluorometer (Sequola-Turner Model 450, supplied by Waterman LabSales, Hillsboro, OR). Details of the analytical methodology have appeared elsewhere[14,15].

### Interference Study

Different sulfur compounds, such as $CH_3SH$ and $C_3H_7SH$ (Fisher Scientific, Nepean, CA, purified grade) have been tested by using the AFHS. The $CH_3SH$ was spiked to a Tedlar bag (SKC, USA) with nitrogen as matrix. The $C_3H_7SH$ was used as its liquid phase. The concentration of $CH_3SH$ in Tedlar bags ranged from 4 ppb to 100 ppm.

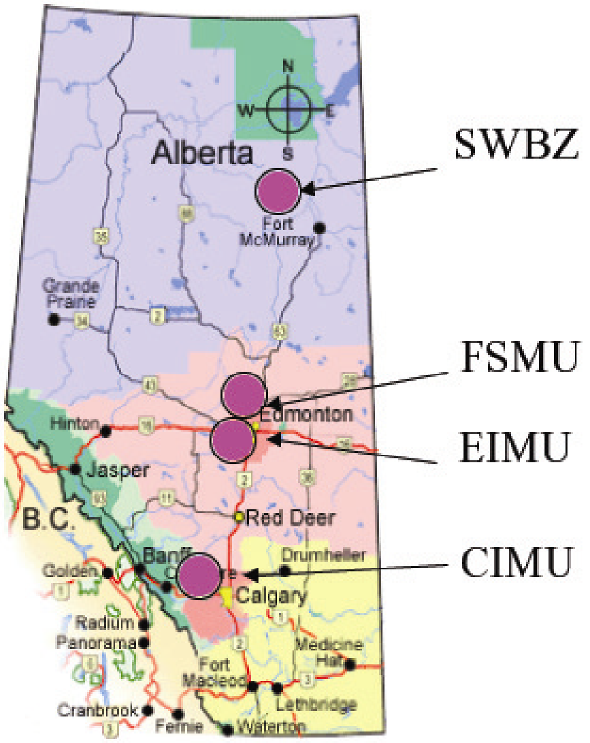

**FIGURE 4.** Field validations in Alberta.

### Field Study

The PASS-HS passive samplers were installed at four locations in Alberta, Canada (Fig. 4). Triplicate passive samplers and duplicate field blanks were used. One location was at the Alberta Environmental Protection (AEP) industrial monitoring site in Edmonton (EIMU), which is equipped with a WSP monitoring device (Wind Flo 540, Athabasca Research Ltd., Edmonton, AB), a temperature measurement device (Model 41372, Campbell Scientific, Inc., Logan, UT), and a RH measurement device (Model 41372, Campbell Scientific, Inc., Logan, UT). Other locations were at Calgary (CIMU), Fort Saskatchewan (FSMU), and Fort McMurray (SWBZ). The exposed time varied from 1 to 15 days. The rain shelters were fastened using the outside bracket to the fittings on the air quality monitoring trailers so that the passive samplers were at the same elevation as the inlet for the continuous analyzers.

## RESULTS AND DISCUSSION

### Laboratory Study

A number of studies were conducted in the Maxxam chamber. The test conditions, measured sampling rates, etc. for several studies are listed in Table 1. The lab triplicate results are very close. Average relative standard deviation was 6%. The measured sampling rates ($R_s$) ranged from 64 to 105 ml/min, which reveals the effects of varying temperature, RH, and WSP on the sampling rate.

**TABLE 1**
**Calculated and Measured $R_S$ Values from Experiments using the Maxxam Chamber**

| | | | | $R_S$ Value (ml/min) | | |
|---|---|---|---|---|---|---|
| **Test No.** | **Temp (ºC)** | **RH (%)** | **WSP (cm/s)** | **Measured** | **Calculated** | **Error (%)** |
| 1 | 1 | 48 | 130 | 90 | 84 | 7 |
| 2 | 20 | 31 | 130 | 105 | 100 | 5 |
| 3 | 20 | 31 | 100 | 99 | 98 | 2 |
| 4 | 20 | 31 | 40 | 94 | 95 | 1 |
| 5 | 20 | 31 | 0.5 | 81 | 92 | 14 |
| 6 | 20 | 66 | 130 | 99 | 96 | 3 |
| 7 | 20 | 84 | 130 | 94 | 95 | 1 |
| 8 | 20 | 30 | 150 | 105 | 100 | 5 |
| 9 | -20 | 50 | 130 | 70 | 64 | 9 |

### *Practical Quantitative Detection Limit*

From laboratory passive sampler filter blank results, it is found that the pooled standard deviation was 12 ng of sulfide per filter based on a 24-h exposure. Thus the practical quantitative detection limit can be taken as 120 ng per filter (10 times the standard deviation). This is equivalent to exposure of the passive sampler to 0.6 ppb $H_2S$ for 24 h. If the exposure period was increased to 1 month (30 days), the method practical quantitative detection limit for $H_2S$ in the atmosphere would be about 0.02 ppb.

The PASS-HS passive samplers were also exposed for 4 days in the Maxxam chamber at 50 ppb, 130 cm/s of WSP, 20°C and 40% of RH. It was observed that the passive samplers were still not saturated. Therefore, based on a 1-month exposure period, it is reasonable to believe that the passive sampler can be used to monitor at least 7 ppb $H_2S$ in the atmosphere. This detection limit satisfies government standards. For example, the Alberta government monthly standard for $H_2S$ is 3 ppb.

### *Effect of Temperature*

It is well known that the collected amount of any air pollutant (Q) is a function of its concentration ($C_e$) in the atmosphere and its diffusion coefficiency (D).

$$Q = f(C_e D) \tag{1}$$

From the ideal gas law, $C_e$ can be calculated as follows:

$$PV = nRT \tag{2}$$

$$\frac{n}{V} = \frac{P}{RT} = C_e \tag{3}$$

D is a function of absolute temperature and pressure.

$$D = f(T^{3/2}/P) \quad (4)$$

Thus Q can be simplified as Eq. 5.

$$Q = f(P/T, T^{3/2}/P) = f(T^{1/2}) \quad (5)$$

Actually, Q is a function of $R_S$. Accordingly, $R_S$ should also be a function of $T^{1/2}$, which was clearly demonstrated in the laboratory studies of the $H_2S$ collection. As shown in Table 1, the sampling rates increase with the increase of the temperature. For example, at a temperature of −20°C with a 50% RH and 130 cm/s of WSP, the measured $R_S$ was 70 ml/min, but at 1°C and 48% RH, the measured $R_S$ was 90 ml/min. The overall $R_S$ change between −20°C to 1°C was 20 ml/min, which is about 30% increase compared with the $R_S$ at −20°C. This change is very significant.

Average monthly temperatures, as determined by Environment Canada over the period 1961–1990[16] vary 30 to 40°C between January to July for Alberta locations; thus, a substantial difference in sampler performance could be expected when operating the PASS-HS in winter and summer in Alberta. If consideration is given to operate in other areas, such as Miami in summer and Yellowknife, Canada, in winter, the sampling rate change will be more substantial.

### Effect of Face Velocity (Wind Speed)

Many authors have reported the effect of WSP to the sampling rates. Harper and Purnell[17] reported that a minimum WSP of 5–10 cm/s was required. Lewis et al.[18] noticed a continuous increase of sampling rate from WSP of 0 to 100 cm/s. Our studies confirmed these discoveries by other authors. But we also noticed that even if the WSP was higher than 100 cm/s for the PASS-HS passive sampler, the sampling rate continuously increased. The threshold WSP was determined from laboratory studies to be about 130 cm/s or 4.68 km/h. It was found that, within the test range of the WSP, the sampling rates were almost proportional to the WSP.

Monthly average wind speeds vary by locations in the world. For example in Alberta, the wind speeds ranged from 6 to 22 km/h according to Environment Canada[16], which are all above the threshold level of 4.68 m/h. Therefore, the wind speeds will not substantially affect the PASS-HS sampling rates when they are used outdoors in many locations if the PASS is properly installed.

### Effect of Relative Humidity

As can be seen in Table 1, the RH affected the PASS-HS passive sampler sampling rates. The sampling rate increased almost proportionally along with the decrease of the RH within the test range at 20°C. The sampling rates of the PASS-HS passive samplers changed from 105 ml/min at 31% RH to 94 ml/min at 84% RH.

It is believed that the decrease of the sampling rate with the increase of RH might be associated with the reaction between water vapor and $H_2S$. It is very possible that the chemical reaction between water and $H_2S$ prevents the free $H_2S$ from passing the diffusion barrier. Further study is needed in order to reveal the correct reason.

Average monthly RH values reported by Environment Canada[16] for Alberta locations ranged from 50 to 75%. A change in RH would result in a slight change of the sampling rates.

### Equation for Calculation of Sampling Rate

From the laboratory chamber and field study results, an equation for calculating the PASS-HS passive sampler rates associated with temperature, RH, and WSP was determined as follows:

$$R_S = 26.66T^{1/2} - 0.1038RH + 0.0633WSP - 361 \qquad (6)$$

where T = average temperature (in degrees K), RH = average relative humidity, and WSP = average wind speed, cm/s. If WSP > 130, then WSP = 130.

From Eq. 6, it can be found that the PASS-HS passive sampler performance was proportional to $26.66T^{1/2}$, $-0.1038RH$, and $0.0633WSP$. Decreases of ambient temperature and wind speed (when WSP < 130) will decrease $R_S$, but increases of relative humidity will result in a decrease in $R_S$.

The calculated values of $R_S$ are shown in Table 1. Compared to the measured $R_S$, it is seen that the lowest accuracy occurred in Test 5, in which the WSP was only 0.5 cm/min or 0.018 km/h. This test further confirmed the observation by other scientists that a minimum 10 cm/s face velocity is required for properly operating a passive sampler. Fortunately, from the average WSP point of view, there are very few cases of such low wind speeds occurring outdoors based on daily or monthly exposure. It is therefore unnecessary to take into consideration such low wind speeds using the PASS-HS passive sampler outdoors.

### Field Study

Field study results are listed in Table 2. Table 2 shows the locations, study periods, meteorological conditions, calculated sampling rates, $H_2S$ concentrations measured by the PASS-HS and the AFHS, and relative errors in each study. Table 3 lists field triplicate passive sampler results. Both tables clearly show that the study accuracy and precision are excellent.

**TABLE 2**
**Field Study Results**

| # | Location | Day | Date | RH % | T ºC | WSP Km/h | $R_S$ cm/min | $H_2S$ ppb Passive | $H_2S$ ppb Active | Error % |
|---|---|---|---|---|---|---|---|---|---|---|
| 1 | EIMU* | 4 | 16–19 March | 48 | 5 | 11 | 87 | 0.92 | 1.11 | 13 |
| 2 | EIMU | 3 | 21–23 March | 68 | −13 | 11 | 70 | 0.44 | 0.44 | 0 |
| 3 | CIMU | 2 | 27–28 March | 57 | 4 | 11 | 85 | 0.35 | 0.31 | 11 |
| 4 | FSMU | 13 | 5–27 April | 54 | 4 | 10 | 85 | 0.20 | 0.20 | 0 |
| 5 | SWBA | 3 | 23–25 May | 65 | 16 | 9 | 94 | 0.40 | 0.42 | 5 |

**TABLE 3**
**Field Triplicate Exposure Results**

| # | Concentrations of $H_2S$ (ppb) | | | Average | STD |
|---|---|---|---|---|---|
| | #1 | #2 | #3 | (ppb) | (%) |
| 1 | 0.94 | 0.89 | 0.92 | 0.92 | 6 |
| 2 | 2.30 | 2.30 | 2.12 | 2.24 | 5 |
| 3 | 1.44 | 1.52 | 1.68 | 1.57 | 6 |
| 4 | 3.15 | 2.70 | 2.88 | 2.90 | 6 |
| 5 | 5.00 | 4.40 | 4.30 | 4.57 | 8 |

The EIMU, CIMU, and FSMU are respectively stations located in the industrial area of Edmonton, Calgary, and Fort Saskatchewan, Alberta, Canada. SWBZ is a station in northern Alberta. Field study results indicate that the relative error using the PASS-HS passive sampler is within 15% compared to the active $H_2S$ sampler

The field study results further prove the importance of Eq. 6. Field Study No. 1 is a typical example. Field Studies No. 1 and No. 2 were conducted at the same location but under different climatic conditions; the calculated sampling rate varied from 87 to 70 ml/min. If the sampling rate obtained in Study No. 1 is used to calculate the $H_2S$ concentration in Study No. 2, the result would be 0.35 ppb. The relative error compared to 0.44 ppb would be 20%. When considering the operation of PASS-HS at different seasons and difference geographical locations, the difference of the sampling rate might be larger.

### Sensitivity of Sampling Rate to Meteorological Conditions

The meteorological parameters used in Eq. 6 can be obtained from local weather forecast stations or Environmental Canada's 30-year weather average (ECN)[14]. It is found that the parameters listed in ECN were close to the parameters monitored by the local weather station (see Table 4). Actually, even a 5°C decrease of temperature can only reduce the sampling rate from 87 to 91 ml/min (Field Study No. 1). The difference is about 4 ml/min. This change is not significant; it can only cause a relative deviation of around 4%. But, at different seasons (such as summer and winter in Alberta) or at different locations in the world (such as Florida and Alberta in winter), the temperature difference might be as great as 50°C. This change is very significant. Assuming RH = 48%, WSP = 12 km/h, a high temperature of 30°C, and a low temperature of −10°C, the sampling rates will change from 106 to 75 ml/min. The difference is 31 ml/min. The relative deviation is about 30% for each individual sampling rate.

The change of RH of 5% will also not seriously affect the sampling rate. Let us still use Field Study No. 1 as an example. If RH changes from 48 to 53%, the $R_S$ changes from 87 to 86 ml/min. The change is only 1 ml/min, which is about 1%. If the RH change is large, such as a change from 20 to 90%, the $R_S$ change will be about 10%.

The CSPSS passive sampler is designed for weekly or monthly monitoring purposes. When meteorological conditions are averaged over a long period in the same season they do not vary greatly within a special geographical area. However, from one geographical area to another, the change may be very significant.

It is concluded that regional average meteorological conditions can be applied to the calculation of $H_2S$ ambient concentration obtained with the passive sampling devices providing these devices are sited according to the general criteria for any air quality monitoring system (no effect from local topography or obstructions). The errors introduced by using regional instead of site-specific meteorological measurement is likely to be less than 10%.

**TABLE 4**
**Comparison of $H_2S$ $R_S$ Obtained between Real Measurement in 2000 and Canadian Climate Normals 1961–1990 (CCN) in Fort St. John**

| Month | Temperature C | | RH % | | $R_S$ H2S ml/min | | |
|---|---|---|---|---|---|---|---|
| | 2000 | CCN | 2000 | CCN | 2000 | CCN | Error % |
| January | −15 | −15 | 70 | 73 | 68 | 68 | 0 |
| February | −10 | −11 | 77 | 70 | 72 | 71 | 0 |
| March | −7 | −6 | 77 | 77 | 74 | 75 | 1 |
| April | 5 | 4 | 60 | 58 | 86 | 85 | 1 |
| May | 8 | 10 | 58 | 55 | 88 | 90 | 2 |
| June | 13 | 14 | 65 | 61 | 91 | 93 | 1 |
| July | 14 | 17 | 67 | 65 | 92 | 94 | 3 |
| August | 16 | 15 | 67 | 70 | 93 | 92 | 1 |
| September | 11 | 10 | 70 | 70 | 89 | 88 | 1 |
| October | 3 | 4 | 64 | 67 | 83 | 84 | 1 |
| November | −3 | −7 | 80 | 76 | 77 | 74 | 4 |
| December | −14 | −13 | 78 | 75 | 68 | 69 | 2 |

**TABLE 5**
**Interference Study Results Using Maxxam Active $H_2S$ Sampler**

| Standard | Samp. Time min | Pump rate ml/min | Measured ng as $H_2S$ | Calculated ng as $H_2S$ |
|---|---|---|---|---|
| Pure $C_3H_7SH$ | 1 drop to filter | | 0 | 1,000,000 |
| 100 ppm $CH_3SH$ | 1 | 500 | 36 | 96,000 |

### Interference

Numerous reagents have been checked for possible interference in this procedure by different authors. Axelrod et al.[14] found no significant interference from sulfate but a small effect from sulfite. Farwell et al.[15] reported that there was no statistically significant interference observed from $SO_2$, $CS_2$, COS, $CH_3SH$, or $CH_3SCH$.

Table 5 summarizes our results. Our studies confirm other groups' findings. Based on the simple chemical reaction between silver nitrate and hydrogen sulfide, only $CH_3SH$ might have stronger interference than other sulfur compounds, since $CH_3SH$ has the same HS group as the $H_2S$ does. But we found almost no interference from $CH_3SH$ and $C_3H_7SH$ in sampling by the active sampler. For example, the amount of $CH_3SH$ collected by the active sampler should be equal to 96,000 ng of $H_2S$. But only 36 ng of $H_2S$ were detected. The ratio is only about 0.0004, which is very insignificant. The Maxxam active sampler and the PASS-HS used the same collection filters. Therefore it is very safe to draw the conclusion that there is almost no significant interference from other sulfur compounds when using PASS-HS to monitor $H_2S$ in air.

### Accuracy

The accuracy of the PASS-HS compared to that of an active sampler can be found in Table 2. The field $H_2S$ concentrations during field studies ranged from 0.02 to 1 ppb. The relative errors between the active and passive samplers ranged from 0 to 12%. Therefore, it is reasonable to conclude that the $H_2S$ concentration measured by the PASS-HS compared to that measured by the active sampler can be higher than 85%.

### APPLICATIONS

The PASS-HS has been used in many air monitoring projects and industrial hygiene studies. The following are two application cases.

***Case 1:*** A local government in Canada monitored $H_2S$ concentrations in ambient air at three locations from October to December 1999. The measured results are shown in Fig. 5. It can be observed that December's $H_2S$ concentration at Location A was very high. Through an investigation, the local authority found that a company near Location A cleaned a storage tank and released a lot of $H_2S$ into the atmosphere.

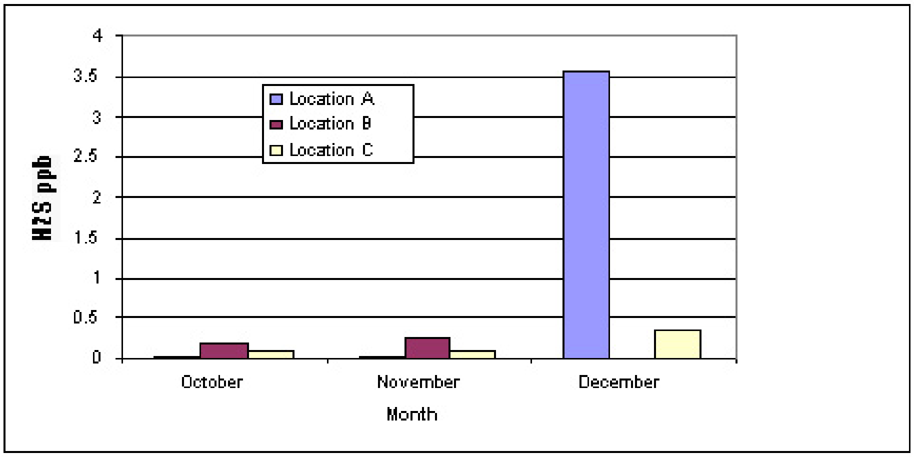

**FIGURE 5.** $H_2S$ concentrations in 3 months at three locations.

***Case 2:*** An oil and gas company planned to operate a new gas well in 2000. The company needed to monitor the impact of $H_2S$ on the local air quality. In order to do so, it first conducted a baseline study before the operation at the well site and in a farmer's back yard. After the operation, the company conducted a 3-month monitoring program. Fig. 6 shows the measurement results. It is clear that the first month's $H_2S$ concentration was too high at the well site due to a leak problem. After fixing the leak problem, the $H_2S$ concentrations were relatively stable.

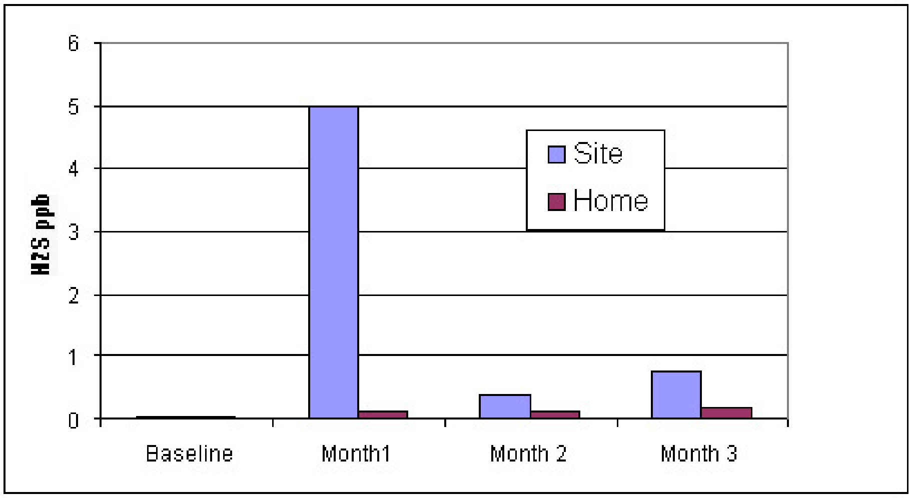

**FIGURE 6.** $H_2S$ concentrations at different locations before and after the operation.

### CONCLUSIONS

The new Maxxam All-Season $H_2S$ Passive Sampling System described in this paper is suitable for monitoring $H_2S$ concentrations in the atmosphere for field studies, especially for networks. The unique design of PASS-HS can avoid the effects of suspended particles in the atmosphere on the sampling rates. The equation determined from the lab and field studies can provide reasonable sampling rates for field measurement. Based on the laboratory study, the PASS-HS can be used to measure ambient $H_2S$ concentrations ranging from 0.02 to 7 ppb for a monthly exposure period.

### ACKNOWLEDGMENTS

Technical support from Ray Brassard and Murray Eliuk of Alberta Environment is highly appreciated.

### BIOSKETCHES

**Hongmao Tang** is Senior Research Scientist and R & D Director in Maxxam Analytics Inc. Canada. He received his M.S. degree from Shandong University (China) and his Ph.D. degree from Brigham Young University (U.S.). His research fields cover environmental and analytical chemistry. He has published more than 90 papers in refereed journals and conference proceedings. For the last several years, he has been concentrating in new passive sampling technology developments for monitoring air pollution. He is principle inventor of Maxxam All-season Passive Sampling System. He received 14 awards from China, U.S., and Canada.

**James Sandeluk** has a B.S. degree. He was Research Assistant in the Centre for Passive Sampling Technology, Maxxam Analytics Inc. Currently he is a student at the University of Alberta, Canada.

**Linda Lin** has an M.S. degree. She is Research Scientist and Laboratory Supervisor in the Centre for Passive Sampling Technology, Maxxam Analytics Inc. She joined the Centre in 2001.

**J. William Lown** received the Ph.D. and D.I.C. degrees in organic chemistry at the Imperial College of Science, Technology and Medicine. He was appointed Professor of Organic Chemistry in University of Alberta Canada in 1974, and was appointed McCalla Research Professor in 1988. Dr. Lown has delivered over 250 invited lectures in North America, Europe, Asia, and South America and has published over 400 research papers in refereed journals, mainly in cancer chemotherapy. Dr. Lown was awarded the Paul Ehrlich Prize for 1992 by the Association de Recherches Scientifiques Paul Neumann. This distinction rewards a scientist or team of scientists who have made in the past 10 years an outstanding contribution in the field of Medicinal Chemistry.
